# Low dose naltrexone: Effects on medication in rheumatoid and seropositive arthritis. A nationwide register-based controlled quasi-experimental before-after study

**DOI:** 10.1371/journal.pone.0212460

**Published:** 2019-02-14

**Authors:** Guttorm Raknes, Lars Småbrekke

**Affiliations:** 1 Regional Medicines Information and Pharmacovigilance Centre (RELIS), University Hospital of North Norway, Tromsø, Norway; 2 Raknes Research, Ulset, Norway; 3 Department of Pharmacy, Faculty of Health Sciences, UiT - The Arctic University of Norway, Tromsø, Norway; Campus Bio-Medico University of Roma, ITALY

## Abstract

In recent years, low dose naltrexone (LDN) has been used as an off-label therapy for several chronic diseases. Results from small laboratory and clinical studies indicate some beneficial effects of LDN in autoimmune diseases, but clinical research on LDN in rheumatic disease is limited. Using a pharmacoepidemiological approach, we wanted to test the hypothesis that starting LDN leads to reduced dispensing of medicines used in the treatment of rheumatic disease. We performed a controlled before-after study based on the Norwegian Prescription Database (NorPD) to compare prescriptions to patients one year before and one year after starting LDN in 2013. The identified patients (n = 360) were stratified into three groups based on LDN exposure. Outcomes were differences in dispensing of medicines used in rheumatic disease. In persistent LDN users, there was a 13% relative reduction in cumulative defined daily doses (DDD) of all medicines examined corresponding to -73.3 DDD per patient (95% CI -120,2 to -26.4, p = 0.003), and 23% reduction of analgesics (-21.6 DDD (95% CI -35.5 to -7.6, p<0.009)). There was no significant DDD change in patients with lower LDN exposure. Persistent LDN users had significantly reduced DDDs of NSAID and opioids, and a lower proportion of users of DMARDs (-6.7 percentage points, 95% CI -12.3 to-1.0, p = 0.028), TNF-α antagonists and opioids. There was a decrease in the number of NSAID users among patients with the least LDN exposure. Important limitations are that prescription data are proxies for clinical effects and that a control group unexposed to LDN is lacking. The results support the hypothesis that persistent use of LDN reduces the need for medication used in the treatment of rheumatic and seropositive arthritis. Randomised clinical trials on LDN in rheumatic disease are warranted.

## Introduction

Some patients, doctors and researchers claim that low dose naltrexone (LDN, typically <5mg/day) is an efficacious alternative off-label therapy in several autoimmune diseases. There are indications that naltrexone interacts with the opioid growth factor receptor (OGFr) on immune cells directly as an antagonist or by modulating the amount of OGFr agonists like metenkephalin [1]. Beneficial effects of LDN have been seen in experimental autoimmune encephalomyelitis (EAE), an animal model of multiple sclerosis (MS) [2,3]. In a small randomized trial in patients with Crohn’s disease, there were improvements in objective histologic and endoscopic measures in the LDN group compared with placebo [4], and small studies indicate effects on some outcome variables in MS [5] and psoriasis [6].

A sudden and large surge in prescribing of LDN in Norway after a TV documentary in 2013 [7] gave us a unique opportunity to study whether initiation of LDN use is associated with changes in the dispensing of relevant medicines [8]. Among MS patients there was no association between starting LDN and drug consumption [9], but we found a decrease in the number of users of several medicines used in inflammatory bowel disease (IBD) [10]. In addition, in the entire LDN-using population, there was a 47% reduction in opioid consumption among persistent LDN users [11].

In spite of autoimmune aetiology and claims of efficacy [12], there is surprisingly little research on LDN in rheumatoid and seropositive arthritis. If efficacious, it is plausible that starting LDN could significantly reduce the need for analgesics and disease-modifying antirheumatic drugs (DMARDs). Data from the Norwegian Prescription Database (NorPD) are well suited to examine possible effects on dispensing of medicines to these patients. The aim of the study is to investigate whether there is an association between LDN exposure and significant changes in the dispensing of relevant medicines in rheumatoid and seropositive arthritis.

## Methods

### Study design and setting

This is a quasi-experimental study with controlled before-after comparisons of the dispensing of medicines in rheumatoid and seropositive arthritis. The design is similar to our previous studies on MS and IBD [9,10].

In short, we used the NorPD to identify and include patients. NorPD contains encrypted information on all prescriptions dispensed to the entire Norwegian population living outside hospitals and nursing homes, and a unique person identity number enabled us to follow dispensing on individual level over time [13]. The Norwegian Institute of Public Health hosts the database. For a fee and after an application according to data access procedures [14], we received a data file of all prescriptions from January 1 2009, to December 31 2015, dispensed to patients who had collected at least one LDN prescription (product identification code 361181) in 2013.

### Study subjects

NorPD contains diagnostic codes for reimbursed dispenses. General practitioners use the International Classification of Primary Care 2 (ICPC-2) [15], and we used the code L88 to identify patients with rheumatoid/seropositive arthritis. This code also covers some allied conditions like ankylosing spondylitis and juvenile arthritis. Psoriatic arthropathy (L99) is an explicit exclusion criterion. In order to avoid bias from newly diagnosed patients, we identified patients from code L88 in two years (2009 and 2010) preceding the observation period (2012 to 2014). To increase specificity, two reimbursed prescriptions with code L88 from both 2009 and 2010 were required for inclusion in the study.

Like in our previous pharmacoepidemiological studies on LDN [9–11], we stratified the patients into three groups based on LDN exposure: LDN ×1 (one LDN prescription dispensed), LDN ×2–3 (two or three LDN prescriptions dispensed) and LDN ×4+ (four or more LDN prescriptions dispensed). The patients served as their own controls (before data) and between groups that reflect LDN exposure. We considered the LDN x 4+ patients as persistent users, compared with the patients in the LDN x 1 who likely used LDN for a much shorter time. The LDN x 2–3 group enables dose-response comparisons.

### Outcome variables

We used the following NorPD variables: Encrypted person identifier, birth year and sex, reimbursement code, Anatomical Therapeutic Chemical Classification (ATC) code, product identifying number, date of dispensing, and dispensed volume in Defined Daily Doses (DDDs). We defined the dispensing date of the first prescription on LDN in 2013 as the index date for each included patient. The outcomes were the differences in dispensing in the one year before compared with the first year after the index date, expressed as average cumulative DDDs and as the number of users in each LDN exposure group.

We defined the primary outcomes as change in cumulative DDDs and number in users of:

- All medicines being studied
- DMARDs: (TNF-α inhibitors + systemic corticosteroids + other DMARDs (aminosalicylates, anakinra, antimalarials, azathioprine, ciclosporin, mercaptopurine, leflunomide, methotrexate, rituximab, tacrolimus, and tocilizumab))
- Non-steroid anti-inflammatory drugs (NSAIDs) (ATC M02A)
- Analgesics (ATC N02: Opioids, and paracetamol and other non-opioid analgesics).

Secondary outcomes were the differences in DDDs and in the number of users of subgroups of main outcome medicines. These were TNF-α inhibitors, systemic corticoids and other DMARDs, opioids and other analgesics. Differences in DDDs and the number of users of the ATC group L04A (immunosuppressants) was a secondary outcome. All outcomes were assessed for difference-in-difference between groups.

### Measurement

For each patient, we summarized the number of collected DDDs and the number of users for all relevant medicines one year (365 days) before and one year after the index date (index date + 364 days). The total observation time was 2 years for all participants. The first observation pre-index date was theoretically January 1, 2012, and the last observation date post-index date was December 31, 2014.

### Statistical considerations

The number of patients in NorPD fulfilling our inclusion criteria determined the study size. We used SPSS 25 and Excel 2013 for data analysis, and all data on DDDs were analyzed on an individual level. We used a pairwise *t*-test to determine the significance of mean changes in the sum of the DDDs per patient in each group for all examined medicines and calculated 95% confidence intervals for difference of means. Change in the number of users was expressed as the proportion of each cohort, together with the 95% confidence interval for the difference of proportion [16]. Daily dispensing data was used to construct curves to illustrate the dispensing of different ATC groups throughout the observation period.

### Ethics

The Regional Committee for Medical and Health Research Ethics of Northern Norway reviewed the study protocol. Due to the encrypted data, the committee concluded that disclosure was not mandatory. The local privacy ombudsman for research at the University Hospital of Northern Norway approved the project. Consent from individual patients is by law not required for research based on NorPD.

## Results

**Fig 1:**
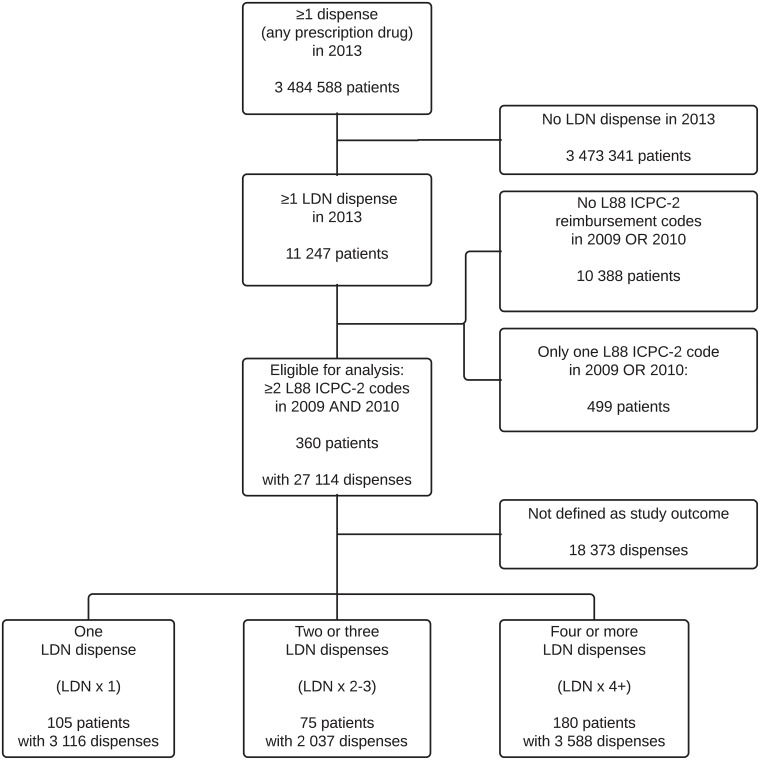
Flowchart showing the inclusion of study subjects and prescription dispenses from the Norwegian Prescription Database (NorPD).

The inclusion of patients and prescription dispenses is shown in Fig 1. We included 360 patients, and the total observation constituted 8640 patient-months. The analyses include 4500 prescriptions dispensed before and 4241 prescriptions dispensed after the LDN index dates.

Table 1 gives baseline data for the three LDN exposure groups. Age and sex distributions were similar. There is a tendency towards less dispensing before starting LDN with increasing LDN exposure.

**Table 1:** Baseline data.

	LDN x 1		LDN x 2–3		LDN x 4+	
**N** (%)	105	(29.2)	75	(20.8)	180	(50.0)
**Female** (%)	83	(79.0)	54	(72.0)	140	(77.8)
**Age** (SD)	60.0	(11.0)	59.8	(9.8)	58.7	(10.5)
**Dispenses per patient one year before index date** (all medicines, SD)	38.9	(44.6)	36.4	(46.9)	34.2	(28.9)

### Main results

Main outcomes are shown in Table 2 (changes in DDDs) and in Table 3 (changes in the number of users). For persistent LDN users (LDN x 4+), there was a significant 13% reduction in the total number of DDDs dispensed of all examined medicines one year after compared with one year before the index date. Among one-time users (LDN x 1) there was a 2% increase in DDDs, but this was not significantly different from zero. There was a significant reduction in the number of users of all medicines being examined in the LDN x 1 group (-4%).

**Table 2:** Average cumulative dose (DDD) of examined medicines dispensed to patients with rheumatoid and seropositive arthritis one year before and after the first dispense of LDN.

		Dispensed medicines (DDD)		Difference (DDD)		p
		Before	After	Mean	95% CI	
**All examined medicines**	LDN x 1	655.7	671.1	15.3	(-49.1 to 79.7)	0.642
	LDN x 2–3	606.9	610.3	3.4	(-90.3 to 97.1)	0.943
	LDN x 4+	558.4	485.1	-73.3	(-120.2 to -26.4)	0.003
**DMARDs**	LDN x 1	287.5	296.8	9.2	(-44.4 to 62.8)	0.737
	LDN x 2–3	254.5	295.9	41.4	(-19.4 to 102.2)	0.186
	LDN x 4+	235.5	215.4	-20.1	(-51.6 to 11.4)	0.213
**NSAIDs**	LDN x 1	184.9	183.1	-1.8	(-27.2 to 23.5)	0.888
	LDN x 2–3	213.4	180.9	-32.5	(-96.6 to 31.7)	0.324
	LDN x 4+	211.0	179.3	-31.6	(-55.0 to -8.3)	0.009
**Analgesics**	LDN x 1	183.3	191.2	7.9	(-11.5 to 27.3)	0.424
	LDN x 2–3	139.0	133.5	-5.5	(-29.9 to 18.8)	0.658
	LDN x 4+	111.9	90.3	-21.6	(-35.5 to -7.6)	0.003
**Corticosteroids**	LDN x 1	94.9	94.3	-0.7	(-17.6 to 16.3)	0.940
	LDN x 2–3	72.4	60.8	-11.6	(-36.7 to 13.5)	0.369
	LDN x 4+	56.1	55.4	-0.8	(-13.0 to 11.5)	0.903
**TNF-α antagonists**	LDN x 1	33.6	29.4	-4.3	(-15.7 to 7.2)	0.467
	LDN x 2–3	33.0	33.9	0.9	(-12.9 to 14.6)	0.902
	LDN x 4+	31.2	25.0	-6.3	(-13.1 to 0.6)	0.076
**Other DMARDs**	LDN x 1	159.0	173.1	14.1	(-26.8 to 55.1)	0.500
	LDN x 2–3	149.1	201.2	52.1	(4.3 to 99.9)	0.036
	LDN x 4+	148.1	135.1	-13.0	(-37.5 to 11.4)	0.297
**Opioids**	LDN x 1	99.4	103.4	4.0	(-8.9 to 17.0)	0.541
	LDN x 2–3	72.7	62.7	-10.0	(-25.4 to 5.5)	0.210
	LDN x 4+	39.7	21.1	-18.6	(-28.1 to -9.0)	<0.001
**Other analgesics**	LDN x 1	83.9	87.8	3.9	(-11.0 to 18.8)	0.609
	LDN x 2–3	66.3	70.7	4.5	(-10.8 to 19.7)	0.569
	LDN x 4+	72.3	69.2	-3.0	(-13.3 to 7.2)	0.562

LDN, low dose naltrexone. DDD, defined daily dose. DMARD, disease-modifying antirheumatic drug. NSAID, a non-steroid anti-inflammatory drug. Three groups based on number of LDN dispenses: LDN ×1 (N = 105) collected LDN once, LDN ×2–3 (N = 75) two or three times and LDN ×4+ (N = 180) four or more times. Other DMARDs include methotrexate, antimalarials, aminosalicylates and leflunomide. DMARDs is the sum of TNF-α antagonists, systemic corticosteroids and other DMARDs. Other analgesics include paracetamol/acetaminophen and other non-opioid analgesics.

**Table 3:** The number of users of examined medicines among patients with rheumatoid and seropositive arthritis one year before and after the first dispense of LDN.

		Number of users				Difference		p
		Before	%	After	%	% points	95%CI	
**All examined medicines**	LDN x 1	104	(99.0)	100	(95.2)	-3.8	(-7.5 to -0.2)	0.050
	LDN x 2–3	70	(93.3)	73	(97.3)	4.0	(-1.8 to 9.8)	0.159
	LDN x 4+	170	(94.4)	164	(91.1)	0.0	(-7.7 to 1.0)	0.128
**DMARD**	LDN x 1	70	(66.7)	69	(65.7)	-1.0	(-9.1 to 7.2)	0.389
	LDN x 2–3	44	(58.7)	48	(64.0)	5.3	(-3.6 to 14.3)	0.202
	LDN x 4+	94	(52.2)	82	(45.6)	-6.7	(-12.3 to -1.0)	0.028
**NSAIDs**	LDN x 1	73	(69.5)	63	(60.0)	-9.5	(-18.1 to -1.0)	0.037
	LDN x 2–3	53	(70.7)	47	(62.7)	-8.0	(-18.3 to 2.3)	0.125
	LDN x 4+	121	(67.2)	114	(63.3)	-3.9	(-10.5 to 2.7)	0.205
**Analgesics**	LDN x 1	79	(75.2)	80	(76.2)	1.0	(-5.2 to 7.1)	0.381
	LDN x 2–3	55	(73.3)	58	(77.3)	4.0	(-7.4 to 15.4)	0.314
	LDN x 4+	115	(63.9)	107	(59.4)	-4.4	(-10.4 to 1.5)	0.136
**Corticosteroids**	LDN x 1	53	(50.5)	53	(50.5)	0.0	(-9.1 to 9.1)	0.399
	LDN x 2–3	25	(33.3)	25	(33.3)	0.0	(-11.1 to 11.1)	0.399
	LDN x 4+	62	(34.4)	54	(30.0)	-4.4	(-10.6 to 1.7)	0.145
**TNF-α antagonists**	LDN x 1	15	(14.3)	12	(11.4)	-2.9	(-7.8 to 2.1)	0.208
	LDN x 2–3	9	(12.0)	11	(14.7)	2.7	(-2.5 to 7.9)	0.240
	LDN x 4+	22	(12.2)	17	(9.4)	-2.8	(-5.2 to -0.4)	0.030
**Other DMARDs**	LDN x 1	42	(40.0)	39	(37.1)	-2.9	(-10.5 to 4.8)	0.306
	LDN x 2–3	29	(38.7)	31	(41.3)	2.7	(-5.6 to 10.9)	0.326
	LDN x 4+	57	(31.7)	45	(25.0)	-6.7	(-10.3 to -3.0)	0.001
**Opioids**	LDN x 1	54	(51.4)	55	(52.4)	1.0	(-6.7 to 8.7)	0.387
	LDN x 2–3	42	(56.0)	39	(52.0)	-4.0	(-15.4 to 7.4)	0.314
	LDN x 4+	77	(42.8)	57	(31.7)	-11.1	(-19.0 to -3.3)	0.008
**Other analgesics**	LDN x 1	60	(57.1)	64	(61.0)	3.8	(-2.1 to 9.7)	0.177
	LDN x 2–3	36	(48.0)	43	(57.3)	9.3	(-1.2 to 19.9)	0.089
	LDN x 4+	88	(48.9)	85	(47.2)	-1.7	(-7.1 to 3.8)	0.333

LDN, low dose naltrexone. DMARD, disease-modifying antirheumatic drug. NSAID, a non-steroid anti-inflammatory drug. Three groups based on number of LDN dispenses: LDN ×1 (N = 105) collected LDN once, LDN ×2–3 (N = 75) two or three times and LDN ×4+ (N = 180) four or more times. Other DMARDs include methotrexate, antimalarials, aminosalicylates and leflunomide. DMARDs is the sum of TNF-α antagonists, systemic corticosteroids and other DMARDs. Other analgesics include paracetamol/acetaminophen and other non-opioid analgesics

### DMARDs

There were no significant changes in cumulative DDD per patient of DMARDs in any group. After starting LDN, there was a significant 13% relative reduction of DMARD users in the LDN x 4+ group.

For other DMARDs, there were no reductions in DDDs, but the LDN x 2–3 group had a 35% relative increase.

**Fig 2:**
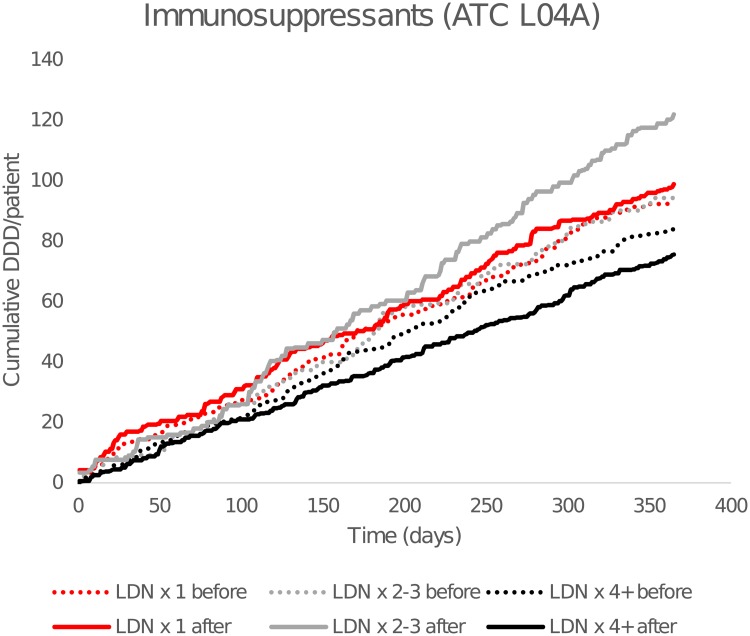
Cumulative dispensed average defined daily doses (DDDs) of immunosuppressants. By time before and after the first low-dose naltrexone (LDN) prescription. Dashed lines show cumulative consumption for the 365 days preceding the first LDN dose, and solid lines after. Anatomical Therapeutic Chemical Classification (ATC) group L04A = immunosuppressants.

Cumulative DDD per patient of ATC group L04A (immunosuppressants) is shown in Fig 2. There was no significant difference in DDD, but the number of users, was reduced by 19% in LDN x 4+ (-5.0% points, 95% CI -8.2 to -1.8, p = 0.003), compared with LDN x 1 (-2.9% points, 95% CI -10.0 to 4.4).

Results for antimalarials, methotrexate, aminosalicylates and leflunomide are presented in S1 and S2 Tables. There were no significant differences in DDDs, but in the LDN x 4+ group, there was a 19% reduction in methotrexate users (-5.0% points, 95% CI -8.2 to -1.8, p = 0.003). Among included patients, there were no users of anakinra, azathioprine, ciclosporin, mercaptopurine, rituximab, tacrolimus, and tocilizumab in any group neither before nor after starting LDN.

**Fig 3:**
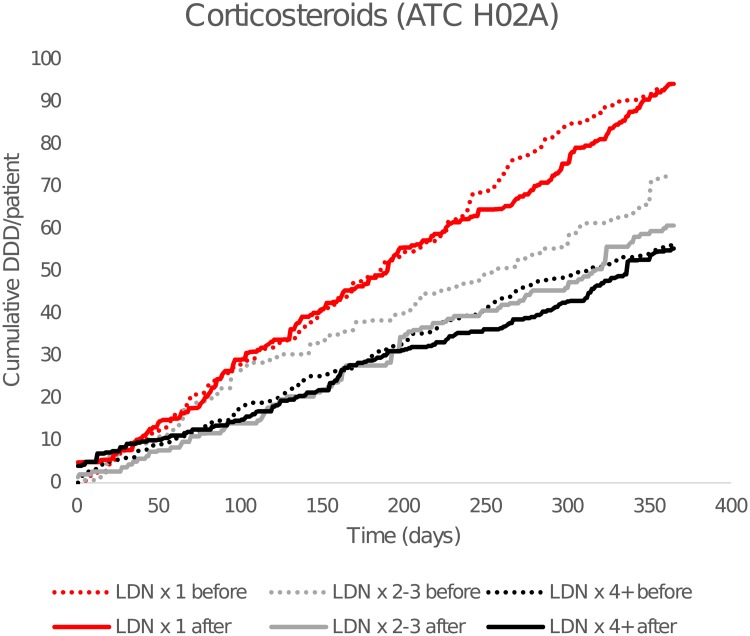
Cumulative dispensed average defined daily doses (DDDs) of corticosteroids. By time before and after the first low-dose naltrexone (LDN) prescription. Dashed lines show cumulative consumption for the 365 days preceding the first LDN dose, and solid lines after. Anatomical Therapeutic Chemical Classification (ATC) group H02A = corticosteroids.

As seen in Fig 3, there was a higher consumption of corticosteroids in the LDN x1 group, but the dispensing was unaffected by LDN (Fig 3). There were reductions in the dispensing of TNF-α-antagonists in both LDN x1 and LDN x4+, but statistical significance was only seen for a relative 23% decrease in the number of users in LDN x4+.

### NSAIDs and analgesics

**Fig 4:**
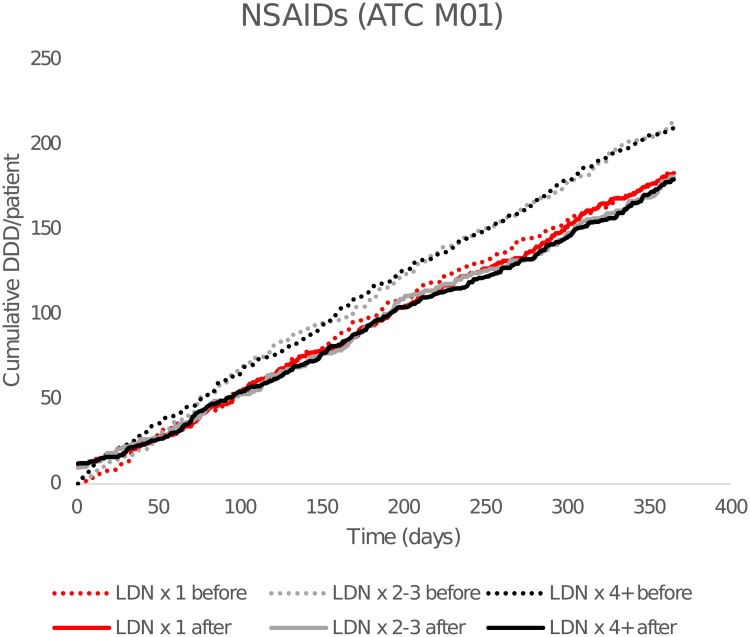
Cumulative dispensed average defined daily doses (DDDs) of NSAIDs. By time before and after the first low-dose naltrexone (LDN) prescription. Dashed lines show cumulative consumption for the 365 days preceding the first LDN dose, and solid lines after. ATC: Anatomical Therapeutic Chemical Classification (ATC) (M02A = NSAIDs).

As seen in Fig 4, there were reductions in cumulative DDDs of NSAID in both LDN x 2–3 and LDN x4+, but the difference was only significant in the LDN x 4+ group (-15%). In the LDN x 1 group, there was a significant 14% reduction in the number of NSAIDs users after starting with LDN.

**Fig 5:**
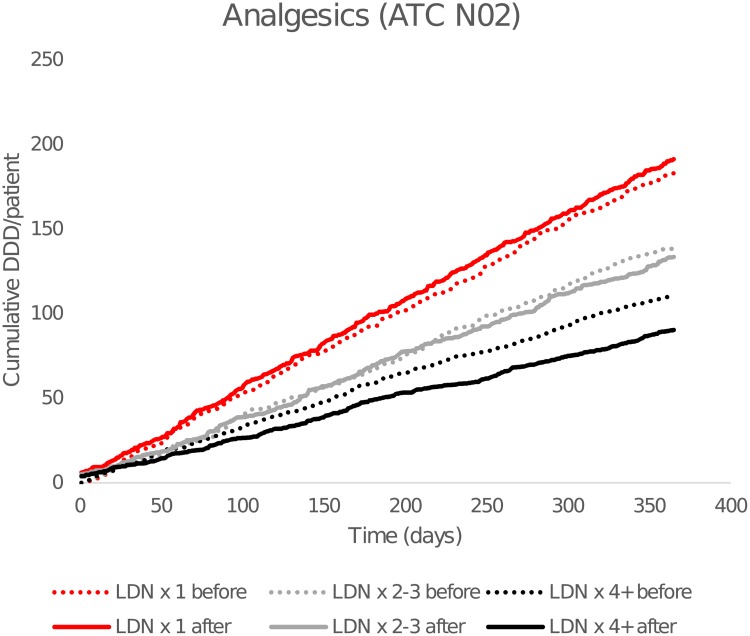
Cumulative dispensed average defined daily doses (DDDs) of analgesics. By time before and after the first low-dose naltrexone (LDN) prescription. Dashed lines show cumulative consumption for the 365 days preceding the first LDN dose, and solid lines after. Anatomical Therapeutic Chemical Classification (ATC) N02 = analgesics (including opioids).

The reduction in cumulative DDDs of analgesics (Fig 5) was significant in the LDN x 4+ group (-19%), but there was no difference in the number of users. The difference is mainly attributable to a reduction in the dispensing of opioids.

Significant difference-in-difference was only observed for cumulative opioid dose in LDN x4+ compared with LDN x1+.

## Discussion

Among persistent LDN users in patients with rheumatic disease, initiation of LDN therapy was followed by significant and clinically relevant reductions in cumulative dispensed dose or in the number of users of all examined medicines; DMARDs including immunosuppressants, NSAIDs and analgesics. Apart from a reduction in the number of users of NSAIDs in patients that collected LDN only once, the use of relevant medication was unaffected in non-persistent LDN users.

The 2013 surge in LDN use in Norway has enabled quasi-experimental pharmacoepidemiological studies. However, such studies have important strengths and limitations. On the favourable side, our study was based on a comprehensive, complete register of all dispenses of prescription medicines to the entire Norwegian population. The observations are real-world-data, in contrast to potential bias in clinical study settings due to strict inclusion criteria or drop-outs. Controlled before-after studies may suggest causal inference, but self-assignment of study subjects makes the design weaker than randomised controlled trials. For example, as seen in S3 Table, there were differences between LDN x 1 and LDN x 4+ groups before starting LDN in the use of DMARDs, NSAIDs and corticosteroids that may reflect different disease activity. By using before-after *differences* in dispensing as outcomes, rather than differences in means only after LDN (as seen in S3 Table), we partly compensate for this potential bias. In addition to comparisons between groups, study participants served as their own controls in a before-after manner. This is accordance with recommendations that analysis in controlled before-after studies should compare the difference in both pre-post change and between intervention and control groups [17]. Alternatively, the dichotomic number-of-users outcomes could be measured as the odds of starting, quitting or continuing the examined medicines (S4 Table), or the odds of using them (S3 Table) after starting LDN therapy. Several of the main findings are confirmed this way. We believe that differences in proportion (in percentage points) of users, is more representative since it accounts for use of the examined medicines in individual patients, both before and after the LDN index date [16].

We did not include a control group of patients unexposed to LDN. The review of the ethical committee and the approval from the privacy ombudsman only allowed inclusion of patients that had collected at least one LDN prescription. Baseline data show only minor differences in age and sex. Although it is impossible to deduce from NorPD how the included patients actually used LDN, it is likely that most patients collecting LDN only once used it for a short time, and they should be considered an appropriate control group to patients that collected LDN x 4+.

We used the ICPC-2 L88 reimbursement code to include patients. It has lower precision than ICD-10 codes, and it is problematic that L88 code is broadly and diffusely defined as “Rheumatoid and seropositive arthritis” by WHO. Mainly seronegative conditions like ankylosing spondylitis and juvenile arthritis are covered by this code. Although more precise diagnoses would have been desirable, we still believe that the L88 code covers sufficiently related diseases to justify the present analyses. The vast majority of dispenses in NorPD are from GPs, and using ICD-10 codes would have reduced the number of included patients and diminished the statistical power of the study. By using strict inclusion criteria, we probably increased specificity in including patients with actual rheumatoid or seropositive arthritis. The high proportion of included patients using DMARDs confirms this. It would be valuable to adjust the analyses by specific rheumatoid arthritis characteristics, such as baseline disease activity, disease duration, or autoantibodies status. In addition, information on remission rates and other direct clinical outcomes is highly relevant. Unfortunately, NorPD does not contain this information.

Although we included 360 patients, which makes this study one of the largest LDN studies in any medical condition so far, we were only able to demonstrate significant difference-in-difference between persistent and short time users (LDN x 1) for cumulative dispensed opioid dose.

The observed changes in prescribing only indirectly indicate improvement or deterioration, but it is plausible that changes in the consumption of the examined medicines are associated with the course of the disease.

The results suggest that persistent LDN use is associated with reduced consumption of relevant and differently acting medicines in rheumatoid/seropositive arthritis. Efficacy of LDN in rheumatic disease cannot be ruled out, and this study is in line with our findings in inflammatory bowel disease, but not in multiple sclerosis where the dispensing was unaffected by LDN. Concomitant use LDN and opioids is often discouraged, which probably partly explain the observed reduction in opioid use in rheumatoid/seropositive arthritis. The reduction was similar to what we have observed in the entire Norwegian LDN using population [18].

We have not identified any study on LDN in rheumatoid or seropositive arthritis. Clinical studies have shown promising results of LDN in fibromyalgia [19,20]. In rheumatoid arthritis, pain is not seldom due to concurrent fibromyalgia or non-inflammatory causes [21]. It is possible that the results, and especially the reductions in analgesic use and NSAIDs, could be attributed to concurrent fibromyalgia. On the other hand, the reductions in the dispensing of immunomodulators indicate that LDN may have a therapeutic effect against rheumatic disease.

## Conclusions and implications

The results of this study suggest that persistent LDN use leads to reduced dispensing of several medicines used in rheumatoid and seropositive arthritis, possibly due to therapeutic effects. Randomised clinical trials should be performed to investigate whether LDN has a place in the treatment of rheumatic disease, either as an alternative or as an add-on to established pharmacotherapy. The expired patent on naltrexone makes commercial studies unlikely, but the low cost and LDNs outstanding safety profile make it an attractive candidate for both patients and those who pay for health services.

## Supporting information

S1 Table Average cumulative dose (DDD) of medicines classified as Other DMARDs, dispensed to patients with rheumatoid and seropositive arthritis one year before and after the first dispense of LDN. (PDF)

S2 Table The number of users of medicines classified as Other DMARDs among patients with rheumatoid and seropositive arthritis one year before and after the first dispense of LDN. (PDF)

S3 Table Odds of being a user of examined medicines one year before and one year after starting LDN, by LDN exposure. (PDF)

S4 Table Odds of starting (use only after LDN), discontinuing (use only before LDN), or continuing (use before and after LDN) the examined medicines, by LDN exposure. (PDF)

## References

[1] Li Z, You Y, Griffin N, Feng J, Shan F. Low-dose naltrexone (LDN): A promising treatment in immune-related diseases and cancer therapy. Int Immunopharmacol. 2018: 61: 178–184. 10.1016/j.intimp.2018.05.020 29885638

[2] Rahn KA, McLaughlin PJ, Zagon IS. Prevention and diminished expression of experimental autoimmune encephalomyelitis by low dose naltrexone (LDN) or opioid growth factor (OGF) for an extended period: therapeutic implications for multiple sclerosis Brain Res. 2011; 1381: 243–53. 10.1016/j.brainres.2011.01.036 21256121

[3] McLaughlin PJ, McHugh DP, Magister MJ, Zagon IS. Endogenous opioid inhibition of proliferation of T and B cell subpopulations in response to immunization for experimental autoimmune encephalomyelitis. BMC Immunol. 2015; 16: 24 10.1186/s12865-015-0093-0 25906771 PMC4407783

[4] Smith JP, Bingaman SI, Ruggiero F, Mauger DT, Mukherjee A, McGovern CO, Zagon IS. Therapy with the opioid antagonist naltrexone promotes mucosal healing in active Crohn’s disease: a randomized placebo-controlled trial. Dig Dis Sci. 2011; 56: 2088–97. 10.1007/s10620-011-1653-7 21380937 PMC3381945

[5] Cree BA, Kornyeyeva E, Goodin DS. Pilot trial of low-dose naltrexone and quality of life in multiple sclerosis. Ann Neurol 2010; 68: 145–50. 10.1002/ana.22006 20695007

[6] Bridgman AC, Kirchhof MG. Treatment of psoriasis vulgaris using low-dose naltrexone. JAAD Case Rep. 2018; 4: 827–9. 10.1016/j.jdcr.2018.06.001 30238048 PMC6143714

[7] Skard K. Unknown medicine LDN gives hope to thousands of patients. Tv2.no. 8 May 2013. http://www.tv2.no/a/5316228. Cited 23 November 2018.

[8] Raknes G, Småbrekke L. A sudden and unprecedented increase in low dose naltrexone (LDN) prescribing in Norway. Patient and prescriber characteristics, and dispense patterns. A drug utilization cohort study. Pharmacoepidemiology and Drug Safety. 2017; 26: 136–42. 10.1002/pds.4110 27670755 PMC5298009

[9] Raknes G, Småbrekke L. Low dose naltrexone in multiple sclerosis: Effects on medication use. A quasi-experimental study. PLoSONE 2017; 12: e0187423 10.1371/journal.pone.0187423 29099849 PMC5669439

[10] Raknes G, Simonsen P, Småbrekke L. The Effect of Low-Dose Naltrexone on Medication in Inflammatory Bowel Disease: A Quasi Experimental Before-and-After Prescription Database Study. J Crohns Colitis. 2018; 12: 677–86. 10.1093/ecco-jcc/jjy008 29385430 PMC5972567

[11] Raknes G, Smabrekke L. Low dose naltrexone and opioid consumption. A drug utilization cohort study based on data from the Norwegian prescription database. Pharmacoepidemiol Drug Saf. 2017; 26: 685–93. 10.1002/pds.4201 28370746 PMC5485080

[12] Shiller AD. Healing Chronic Pain and Illness with LDN (Low Dose Naltrexone) Part 1. 16 August 2017. https://www.drshiller.com/healing-chronic-pain-illness-ldn-low-dose-naltrexone-part-1/. Cited 23 November 2018.

[13] Furu K. Establishment of the nationwide Norwegian Prescription Database (NorPD)–new opportunities for research in pharmacoepidemiology in Norway. Nor J Epidemiol. 2008; 18: 129–36. 10.5324/nje.v18i2.23

[14] Norwegian Institute of Public Health. Norwegian Prescription Database. 20 September 2015. https://www.fhi.no/en/hn/health-registries/norpd/norwegian-prescription-database/. Cited 23 November 2018.

[15] Classification Committee of the World Organization of Family doctors. ICPC-2: International Classification of Primary Care. 2nd edn Oxford: Oxford University Press, 1998.

[16] Altman DG. Comparing groups—categorical data In: Altman DG, editor. Practical Statistics for Medical Research 1ed London: Chapman & Hall; 1991 p. 234–1.

[17] Polus S, Pieper D, Burns J, Fretheim A, Ramsay C, Higgins JPT, Mathes T, Pfadenhauer LM, Rehfuess EA. Heterogeneity in application, design, and analysis characteristics was found for controlled before-after and interrupted time series studies included in Cochrane reviews. J Clin Epidemiol. 2017;91: 56–69. 10.1016/j.jclinepi.2017.07.008 28750849

[18] Raknes G, Småbrekke L. Low-dose naltrexone and opioid consumption: a drug utilization cohort study based on data from the Norwegian prescription database. Pharmacoepidemiol Drug Saf. 2017; 26: 685–93. 10.1002/pds.4201 28370746 PMC5485080

[19] Younger J, Mackey S. Fibromyalgia symptoms are reduced by low-dose naltrexone: a pilot study. Pain med. 2009; 10: 663–72. 10.1111/j.1526-4637.2009.00613.x 19453963 PMC2891387

[20] Younger J, Noor N, McCue R, Mackey S. Low‐dose naltrexone for the treatment of fibromyalgia: findings of a small, randomized, double‐blind, placebo‐controlled, counterbalanced, crossover trial assessing daily pain levels. Arthritis Rheum. 2013; 65: 529–38. 10.1002/art.37734 23359310

[21] Boyden SD, Hossain IN, Wohlfahrt A, Lee YC. Non-inflammatory Causes of Pain in Patients with Rheumatoid Arthritis. Curr Rheumatol Rep. 2016; 18: 30 10.1007/s11926-016-0581-0 27097817

