# Correction: Low dose naltrexone: Effects on medication in rheumatoid and seropositive arthritis. A nationwide register-based controlled quasi-experimental before-after study

**DOI:** 10.1371/journal.pone.0223545

**Published:** 2019-10-01

**Authors:** Guttorm Raknes, Lars Småbrekke

There are a number of errors in Table 3. The value in column 7 "% points" for the line “LDN x 4+” in the group “All examined medicines” should be -3.3, not 0. Additionally, the p-values in the ninth column “P” are incorrect. Please see the correct Table 3 here.

**Table 3:** The number of users of examined medicines among patients with rheumatoid and seropositive arthritis one year before and after the first dispense of LDN.

		Number of users				Difference		
		Before	%	After	%	% points	95% CI	p
**All examined medicines**	LDN x 1	104	(99.0)	100	(95.2)	-3.8	(-7.5 to -0.2)	0.048
	LDN x 2–3	70	(93.3)	73	(97.3)	4.0	(-1.8 to 9.8)	0.184
	LDN x 4+	170	(94.4)	164	(91.1)	-3.3	(-7.7 to 1.0)	0.135
**DMARD**	LDN x 1	70	(66.7)	69	(65.7)	-1.0	(-9.1 to 7.2)	0.819
	LDN x 2–3	44	(58.7)	48	(64.0)	5.3	(-3.6 to 14.3)	0.252
	LDN x 4+	94	(52.2)	82	(45.6)	-6.7	(-12.3 to -1.0)	0.025
**NSAIDs**	LDN x 1	73	(69.5)	63	(60.0)	-9.5	(-18.1 to -1.0)	0.035
	LDN x 2–3	53	(70.7)	47	(62.7)	-8.0	(-18.3 to 2.3)	0.138
	LDN x 4+	121	(67.2)	114	(63.3)	-3.9	(-10.5 to 2.7)	0.251
**Analgesics**	LDN x 1	79	(75.2)	80	(76.2)	1.0	(-5.2 to 7.1)	0.764
	LDN x 2–3	55	(73.3)	58	(77.3)	4.0	(-7.4 to 15.4)	0.493
	LDN x 4+	115	(63.9)	107	(59.4)	-4.4	(-10.4 to 1.5)	0.146
**Corticosteroids**	LDN x 1	53	(50.5)	53	(50.5)	0.0	(-9.1 to 9.1)	1.000
	LDN x 2–3	25	(33.3)	25	(33.3)	0.0	(-11.1 to 11.1)	1.000
	LDN x 4+	62	(34.4)	54	(30.0)	-4.4	(-10.6 to 1.7)	0.159
**TNF-α antagonists**	LDN x 1	15	(14.3)	12	(11.4)	-2.9	(-7.8 to 2.1)	0.259
	LDN x 2–3	9	(12.0)	11	(14.7)	2.7	(-2.5 to 7.9)	0.321
	LDN x 4+	22	(12.2)	17	(9.4)	-2.8	(-5.2 to -0.4)	0.027
**Other DMARDs**	LDN x 1	42	(40.0)	39	(37.1)	-2.9	(-10.5 to 4.8)	0.468
	LDN x 2–3	29	(38.7)	31	(41.3)	2.7	(-5.6 to 10.9)	0.529
	LDN x 4+	57	(31.7)	45	(25.0)	-6.7	(-10.3 to -3.0)	0.001
**Opioids**	LDN x 1	54	(51.4)	55	(52.4)	1.0	(-6.7 to 8.7)	0.809
	LDN x 2–3	42	(56.0)	39	(52.0)	-4.0	(-15.4 to 7.4)	0.493
	LDN x 4+	77	(42.8)	57	(31.7)	-11.1	(-19.0 to -3.3)	0.007
**Other analgesics**	LDN x 1	60	(57.1)	64	(61.0)	3.8	(-2.1 to 9.7)	0.209
	LDN x 2–3	36	(48.0)	43	(57.3)	9.3	(-1.2 to 19.9)	0.094
	LDN x 4+	88	(48.9)	85	(47.2)	-1.7	(-7.1 to 3.8)	0.549

LDN, low dose naltrexone. DMARD, disease-modifying antirheumatic drug. NSAID, a non-steroid anti-inflammatory drug. Three groups based on number of LDN dispenses: LDN ×1 (*N* = 105) collected LDN once, LDN ×2–3 (*N* = 75) two or three times and LDN ×4+ (*N* = 180) four or more times. Other DMARDs include methotrexate, antimalarials, aminosalicylates and leflunomide. DMARDs is the sum of TNF-α antagonists, systemic corticosteroids and other DMARDs. Other analgesics include paracetamol/acetaminophen and other non-opioid analgesics.

There are a number of errors in S2 Table. The p-values in the ninth column “P” are incorrect. Please view the correct S2 Table below.

## Supporting information

S2 Table The number of users of medicines classified as Other DMARDs among patients with rheumatoid and seropositive arthritis one year before and after the first dispense of LDN. (PDF)
