# Supplementary material for: Low dose naltrexone: Effects on medication in rheumatoid and seropositive arthritis. A nationwide register-based controlled quasi-experimental before-after study
Source: PLoS One. 2019 Feb 14;14(2):e0212460. doi: 10.1371/journal.pone.0212460 (PMC6375629; doi:10.1371/journal.pone.0212460)
Supplement: S1 Table — (PDF) [file pone.0212460.s001.pdf]

S1 Table: Average cumulative dose (DDD) of drugs classified as Other DMARDs, dispensed to patients with rheumatoid and seropositive arthritis one year before and after the first dispense of LDN.

	LDN exposure	Dispensed drugs (DDD)		Difference (DDD)		
		Before	After	Mean	95% CI	p
**All other DMARDs**	LDN x 1	159.0	173.1	14.1	(-26.8 to 55.1)	0.500
	LDN x 2-3	149.1	201.2	52.1	(4.3 to 99.9)	0.036
	LDN x 4+	148.1	135.1	-13.0	(-37.5 to 11.4)	0.297
**Antimalarials**	LDN x 1	49.5	49.5	0.0	(-13.5 to 13.5)	1.000
	LDN x 2-3	51.2	65.3	14.1	(-3.5 to 31.8)	0.120
	LDN x 4+	43.9	40.6	-3.3	(-15.6 to 8.9)	0.594
**Methotrexate**	LDN x 1	93.2	98.8	5.6	(-19.2 to 30.4)	0.657
	LDN x 2-3	94.2	121.9	27.7	(-0.7 to 56)	0.059
	LDN x 4+	84.8	75.5	-9.3	(-22.1 to 3.6)	0.158
**Aminosalicylates**	LDN x 1	13.0	14.3	1.4	(-2.6 to 5.4)	0.505
	LDN x 2-3	3.0	3.3	0.3	(-0.8 to 1.5)	0.567
	LDN x 4+	12.8	12.9	0.1	(-7.2 to 7.4)	0.973
**Leflunomide**	LDN x 1	3.3	10.5	7.1	(-3.7 to 18)	0.198
	LDN x 2-3	0.7	10.7	10.0	(-2.2 to 22.2)	0.112
	LDN x 4+	6.7	6.1	-0.6	(-3.4 to 2.3)	0.707

LDN, low dose naltrexone. DDD, defined daily dose. DMARD, disease modifying antirheumatic drug. Three groups based on number of LDN dispenses: LDN ×1 ($N = 164$) collected LDN once, LDN ×2–3 ($N = 115$) two or three times and LDN ×4+ ($N = 224$) four or more times. There were no users of anakinra, azathioprine, ciclosporine, mercaptopurine, rituximab, tacrolimus, and tocilizumab neither before nor after starting LDN
