# Supplementary material for: Low dose naltrexone: Effects on medication in rheumatoid and seropositive arthritis. A nationwide register-based controlled quasi-experimental before-after study
Source: PLoS One. 2019 Feb 14;14(2):e0212460. doi: 10.1371/journal.pone.0212460 (PMC6375629; doi:10.1371/journal.pone.0212460)
Supplement: S2 Table — (PDF) [file pone.0212460.s002.pdf]

S2 Table: Number of users of drugs classified as Other DMARDs among patients with rheumatoid and seropositive arthritis one year before and after the first dispense of LDN.

	LDN exposure	Number of users				Difference		
		Before	%	After	%	% points	95% CI	p
**Any other DMARDs**	LDN x 1	42	(40.0)	39	(37.1)	-2.9	(-10.5 to 4.8)	0.306
	LDN x 2-3	29	(38.7)	31	(41.3)	2.7	(-5.6 to 10.9)	0.326
	LDN x 4+	57	(31.7)	45	(25.0)	-6.7	(-10.3 to -3.0)	0.001
**Antimalarials**	LDN x 1	23	(21.9)	23	(21.9)	0.0	(-5.3 to 5.3)	0.399
	LDN x 2-3	21	(28.0)	18	(24.0)	-4.0	(-9.8 to 1.8)	0.159
	LDN x 4+	29	(16.1)	23	(12.8)	-3.3	(-6.7 to 0.1)	0.064
**Methotrexate**	LDN x 1	39	(37.1)	36	(34.3)	-2.9	(-10.1 to 4.4)	0.295
	LDN x 2-3	29	(38.7)	30	(40.0)	1.3	(-6.5 to 9.2)	0.377
	LDN x 4+	48	(26.7)	39	(21.7)	-5.0	(-8.2 to -1.8)	0.003
**Aminosalicylates**	LDN x 1	6	(5.7)	5	(4.8)	-1.0	(-4.2 to 2.3)	0.338
	LDN x 2-3	2	(2.7)	3	(4.0)	1.3	(-3.2 to 5.8)	0.337
	LDN x 4+	10	(5.6)	7	(3.9)	-1.7	(-4.1 to 0.8)	0.161
**Leflunomide**	LDN x 1	2	(1.9)	3	(2.9)	1.0	(-2.3 to 4.2)	0.338
	LDN x 2-3	1	(1.3)	3	(4.0)	2.7	(-2.5 to 7.9)	0.240
	LDN x 4+	4	(2.2)	5	(2.8)	0.6	(-0.5 to 1.6)	0.241

LDN, low dose naltrexone. DMARD, disease modifying antirheumatic drug. Three groups based on number of LDN dispenses: LDN ×1 ($N = 105$) collected LDN once, LDN ×2–3 ($N = 75$) two or three times and LDN ×4+ ($N = 180$) four or more times. There were no users of anakinra, azathioprine, ciclosporin, mercaptopurine, rituximab, tacrolimus, and tocilizumab neither before nor after starting LDN.
