# Supplementary material for: Low dose naltrexone: Effects on medication in rheumatoid and seropositive arthritis. A nationwide register-based controlled quasi-experimental before-after study
Source: PLoS One. 2019 Feb 14;14(2):e0212460. doi: 10.1371/journal.pone.0212460 (PMC6375629; doi:10.1371/journal.pone.0212460)
Supplement: S3 Table — (PDF) [file pone.0212460.s003.pdf]

S3 Table: Odds of being a user of examined drugs one year before and one year after starting LDN, by LDN exposure.

		Odds being a user **before** starting LDN			Odds being a user **after** starting LDN		
		**OR**	**95% CI**	**P**	**OR**	**95% CI**	**P**
**Any examined drug**	LDN **x 2-3** vs LDN **x 1**	0.13	(0.02 to 1.18)	0.035	1.83	(0.34 to 9.67)	0.473
	LDN **x 4+** vs LDN **x 1**	0.16	(0.02 to 1.30)	0.052	0.51	(0.18 to 1.44)	0.198
	LDN **x 4+** vs LDN **x 2-3**	1.21	(0.40 to 3.68)	0.731	0.28	(0.06 to 1.25)	0.077
**DMARDs**	LDN **x 2-3** vs LDN **x 1**	0.71	(0.38 to 1.31)	0.272	0.93	(0.50 to 1.72)	0.812
	LDN **x 4+** vs LDN **x 1**	0.55	(0.33 to 0.90)	0.017	0.44	(0.27 to 0.72)	0.001
	LDN **x 4+** vs LDN **x 2-3**	0.77	(0.45 to 1.33)	0.347	0.47	(0.27 to 0.82)	0.007
**NSAIDs**	LDN **x 2-3** vs LDN **x 1**	1.06	(0.55 to 2.02)	0.869	1.12	(0.61 to 2.06)	0.717
	LDN **x 4+** vs LDN **x 1**	0.90	(0.53 to 1.51)	0.688	1.15	(0.70 to 1.89)	0.576
	LDN **x 4+** vs LDN **x 2-3**	0.85	(0.47 to 1.53)	0.590	1.03	(0.59 to 1.80)	0.920
**Analgesics**	LDN **x 2-3** vs LDN **x 1**	0.91	(0.46 to 1.78)	0.773	1.07	(0.53 to 2.15)	0.858
	LDN **x 4+** vs LDN **x 1**	0.58	(0.34 to 1.00)	0.047	0.46	(0.27 to 0.79)	0.004
	LDN **x 4+** vs LDN **x 2-3**	0.64	(0.35 to 1.17)	0.145	0.43	(0.23 to 0.80)	0.006
**TNF- α antagonists**	LDN **x 2-3** vs LDN **x 1**	0.82	(0.34 to 1.98)	0.657	1.33	(0.55 to 3.20)	0.521
	LDN **x 4+** vs LDN **x 1**	0.84	(0.41 to 1.69)	0.617	0.81	(0.37 to 1.77)	0.593
	LDN **x 4+** vs LDN **x 2-3**	1.02	(0.45 to 2.33)	0.961	0.61	(0.27 to 1.37)	0.224
**Systemic corticosteroids**	LDN **x 2-3** vs LDN **x 1**	0.49	(0.27 to 0.91)	0.022	0.49	(0.27 to 0.91)	0.022
	LDN **x 4+** vs LDN **x 1**	0.52	(0.32 to 0.84)	0.008	0.42	(0.26 to 0.69)	0.001
	LDN **x 4+** vs LDN **x 2-3**	1.05	(0.59 to 1.86)	0.865	0.86	(0.48 to 1.53)	0.600
**Other DMARDs**	LDN **x 2-3** vs LDN **x 1**	0.95	(0.52 to 1.74)	0.857	1.19	(0.65 to 2.19)	0.570
	LDN **x 4+** vs LDN **x 1**	0.70	(0.42 to 1.15)	0.154	0.56	(0.34 to 0.95)	0.030
	LDN **x 4+** vs LDN **x 2-3**	0.74	(0.42 to 1.29)	0.281	0.47	(0.27 to 0.84)	0.009
**Opioids**	LDN **x 2-3** vs LDN **x 1**	1.20	(0.66 to 2.18)	0.544	0.98	(0.54 to 1.78)	0.960
	LDN **x 4+** vs LDN **x 1**	0.71	(0.44 to 1.14)	0.157	0.42	(0.26 to 0.69)	0.001
	LDN **x 4+** vs LDN **x 2-3**	0.59	(0.34 to 1.01)	0.054	0.43	(0.25 to 0.74)	0.002
**Other analgesics**	LDN **x 2-3** vs LDN **x 1**	0.69	(0.38 to 1.26)	0.225	0.86	(0.47 to 1.57)	0.626
	LDN **x 4+** vs LDN **x 1**	0.72	(0.44 to 1.16)	0.179	0.57	(0.35 to 0.93)	0.025
	LDN **x 4+** vs LDN **x 2-3**	1.04	(0.60 to 1.78)	0.897	0.67	(0.39 to 1.15)	0.141

LDN, low dose naltrexone. OR: Odds Ratio. DMARD, disease modifying antirheumatic drug. NSAID, non-steroid anti-inflammatory drug. Three groups based on number of LDN dispenses: LDN ×1 (N = 105) collected LDN once, LDN ×2–3 (N = 75) two or three times and LDN ×4+ (N = 180) four or more times. Other DMARDs include methotrexate, antimalarials, aminosalicylates and leflunomide. DMARDs is the sum of TNF-α antagonists, systemic corticosteroids and other DMARDs. Other analgesics include paracetamol/acetaminophen and other non-opioid analgesics.
