# Supplementary material for: Low dose naltrexone: Effects on medication in rheumatoid and seropositive arthritis. A nationwide register-based controlled quasi-experimental before-after study
Source: PLoS One. 2019 Feb 14;14(2):e0212460. doi: 10.1371/journal.pone.0212460 (PMC6375629; doi:10.1371/journal.pone.0212460)
Supplement: S4 Table — (PDF) [file pone.0212460.s004.pdf]

S4 Table. Odds of starting (use only after LDN), discontinuing (use only before LDN), or continuing (use before and after LDN) the examined drugs, by LDN exposure.

		Odds of **starting** drug after LDN			Odds of **discontinuing** drug after LDN			Odds of **continuing** drug after LDN		
		**OR**	**95% CI**	**P**	**OR**	**95% CI**	**P**	**OR**	**95% CI**	**P**
**Any examined drug**	LDN **x 2-3** vs LDN **x 1**	NA	NA	0.017	0.34	(0.04 to 3.12)	0.319	0.58	(0.17 to 1.96)	0.371
	LDN **x 4+** vs LDN **x 1**	NA	NA	0.085	1.64	(0.51 to 5.30)	0.401	0.38	(0.14 to 1.04)	0.051
	LDN **x 4+** vs LDN **x 2-3**	0.51	(0.13 to 1.94)	0.314	4.82	(0.61 to 37.99)	0.101	0.66	(0.25 to 1.70)	0.386
**DMARDs**	LDN **x 2-3** vs LDN **x 1**	1.27	(0.47 to 3.47)	0.636	0.54	(0.16 to 1.78)	0.301	0.86	(0.47 to 1.56)	0.612
	LDN **x 4+** vs LDN **x 1**	0.50	(0.19 to 1.33)	0.156	1.19	(0.53 to 2.64)	0.674	0.52	(0.32 to 0.85)	0.009
	LDN **x 4+** vs LDN **x 2-3**	0.39	(0.14 to 1.08)	0.062	2.22	(0.73 to 6.73)	0.150	0.61	(0.36 to 1.05)	0.074
**NSAIDs**	LDN **x 2-3** vs LDN **x 1**	1.18	(0.35 to 4.01)	0.793	0.96	(0.42 to 2.20)	0.916	1.07	(0.59 to 1.95)	0.820
	LDN **x 4+** vs LDN **x 1**	1.50	(0.56 to 3.99)	0.414	0.77	(0.39 to 1.55)	0.470	1.03	(0.63 to 1.67)	0.907
	LDN **x 4+** vs LDN **x 2-3**	1.27	(0.45 to 3.64)	0.652	0.81	(0.37 to 1.77)	0.596	0.96	(0.56 to 1.65)	0.884
**Analgesics**	LDN **x 2-3** vs LDN **x 1**	2.84	(1.00 to 8.05)	0.043	2.39	(0.75 to 7.61)	0.131	0.70	(0.38 to 1.32)	0.271
	LDN **x 4+** vs LDN **x 1**	1.07	(0.39 to 2.99)	0.891	2.36	(0.85 to 6.52)	0.089	0.48	(0.29 to 0.80)	0.004
	LDN **x 4+** vs LDN **x 2-3**	0.38	(0.16 to 0.92)	0.027	0.99	(0.41 to 2.37)	0.979	0.68	(0.39 to 1.18)	0.171
**TNF-α antagonists**	LDN **x 2-3** vs LDN **x 1**	2.15	(0.35 to 13.17)	0.399	0.27	(0.03 to 2.36)	0.206	1.13	(0.43 to 3.03)	0.801
	LDN **x 4+** vs LDN **x 1**	0.00	NA	0.063	0.57	(0.16 to 2.02)	0.380	0.99	(0.44 to 2.25)	0.982
	LDN **x 4+** vs LDN **x 2-3**	0.00	NA	0.007	2.11	(0.24 to 18.41)	0.488	0.87	(0.36 to 2.12)	0.765
**Systemic corticosteroids**	LDN **x 2-3** vs LDN **x 1**	1.06	(0.42 to 2.65)	0.906	1.06	(0.42 to 2.65)	0.906	0.42	(0.22 to 0.83)	0.012
	LDN **x 4+** vs LDN **x 1**	0.55	(0.24 to 1.28)	0.163	0.97	(0.45 to 2.07)	0.935	0.48	(0.28 to 0.80)	0.005
	LDN **x 4+** vs LDN **x 2-3**	0.52	(0.21 to 1.30)	0.158	0.92	(0.40 to 2.12)	0.839	1.12	(0.58 to 2.15)	0.728
**Other DMARDs**	LDN **x 2-3** vs LDN **x 1**	1.22	(0.39 to 3.78)	0.733	0.54	(0.16 to 1.78)	0.301	1.14	(0.6 to 2.15)	0.685
	LDN **x 4+** vs LDN **x 1**	0.00	NA	<0.001	0.68	(0.28 to 1.63)	0.383	0.76	(0.45 to 1.3)	0.315
	LDN **x 4+** vs LDN **x 2-3**	0.00	NA	<0.001	1.27	(0.40 to 4.07)	0.689	0.67	(0.37 to 1.20)	0.174
**Opioids**	LDN **x 2-3** vs LDN **x 1**	1.27	(0.47 to 3.47)	0.636	2.08	(0.79 to 5.46)	0.129	0.90	(0.50 to 1.65)	0.741
	LDN **x 4+** vs LDN **x 1**	1.11	(0.48 to 2.59)	0.805	3.14	(1.40 to 7.03)	0.004	0.37	(0.22 to 0.62)	<0.001
	LDN **x 4+** vs LDN **x 2-3**	0.87	(0.36 to 2.12)	0.765	1.51	(0.72 to 3.14)	0.273	0.41	(0.23 to 0.72)	0.002
**Other analgesics**	LDN **x 2-3** vs LDN **x 1**	2.67	(1.00 to 7.14)	0.045	2.43	(0.56 to 10.49)	0.221	0.59	(0.33 to 1.08)	0.087
	LDN **x 4+** vs LDN **x 1**	0.91	(0.34 to 2.43)	0.852	2.87	(0.80 to 10.22)	0.091	0.59	(0.36 to 0.96)	0.031
	LDN **x 4+** vs LDN **x 2-3**	0.34	(0.14 to 0.81)	0.012	1.18	(0.41 to 3.40)	0.758	0.99	(0.57 to 1.71)	0.974

LDN, low dose naltrexone. OR: Odds Ratio. DMARD, disease modifying antirheumatic drug. NSAID, non-steroid anti-inflammatory drug. Three groups based on number of LDN dispenses: LDN ×1 (N = 105) collected LDN once, LDN ×2–3 (N = 75) two or three times and LDN ×4+ (N = 180) four or more times. Other DMARDs include methotrexate, antimalarials, aminosalicylates and leflunomide. DMARDs is the sum of TNF-α antagonists, systemic corticosteroids and other DMARDs. Other analgesics include paracetamol/acetaminophen and other non-opioid analgesics. NA, not possible to calculate per definition.
