# Supplementary material for: Correction: Low dose naltrexone: Effects on medication in rheumatoid and seropositive arthritis. A nationwide register-based controlled quasi-experimental before-after study
Source: PLoS One. 2019 Oct 1;14(10):e0223545. doi: 10.1371/journal.pone.0223545 (PMC6772034; doi:10.1371/journal.pone.0223545)
Supplement: S2 Table — (PDF) [file pone.0223545.s001.pdf]

**S2 Table: Number of users of drugs classified as Other DMARDs among patients with rheumatoid and seropositive arthritis one year before and after the first dispense of LDN.**

| | LDN exposure | Number of users | | | | Difference | | |
|---|---|---|---|---|---|---|---|---|
| | | Before | % | After | % | % points | 95% CI | p |
| **Any other DMARDs** | LDN x 1 | 42 | (40.0) | 39 | (37.1) | -2.9 | (-10.5 to 4.8) | 0.468 |
| | LDN x 2-3 | 29 | (38.7) | 31 | (41.3) | 2.7 | (-5.6 to 10.9) | 0.529 |
| | LDN x 4+ | 57 | (31.7) | 45 | (25.0) | -6.7 | (-10.3 to -3.0) | 0.001 |
| **Antimalarials** | LDN x 1 | 23 | (21.9) | 23 | (21.9) | 0.0 | (-5.3 to 5.3) | 1.000 |
| | LDN x 2-3 | 21 | (28.0) | 18 | (24.0) | -4.0 | (-9.8 to 1.8) | 0.184 |
| | LDN x 4+ | 29 | (16.1) | 23 | (12.8) | -3.3 | (-6.7 to 0.1) | 0.059 |
| **Methotrexate** | LDN x 1 | 39 | (37.1) | 36 | (34.3) | -2.9 | (-10.1 to 4.4) | 0.440 |
| | LDN x 2-3 | 29 | (38.7) | 30 | (40.0) | 1.3 | (-6.5 to 9.2) | 0.740 |
| | LDN x 4+ | 48 | (26.7) | 39 | (21.7) | -5.0 | (-8.2 to -1.8) | 0.003 |
| **Aminosalicylates** | LDN x 1 | 6 | (5.7) | 5 | (4.8) | -1.0 | (-4.2 to 2.3) | 0.565 |
| | LDN x 2-3 | 2 | (2.7) | 3 | (4.0) | 1.3 | (-3.2 to 5.8) | 0.565 |
| | LDN x 4+ | 10 | (5.6) | 7 | (3.9) | -1.7 | (-4.1 to 0.8) | 0.181 |
| **Leflunomide** | LDN x 1 | 2 | (1.9) | 3 | (2.9) | 1.0 | (-2.3 to 4.2) | 0.565 |
| | LDN x 2-3 | 1 | (1.3) | 3 | (4.0) | 2.7 | (-2.5 to 7.9) | 0.321 |
| | LDN x 4+ | 4 | (2.2) | 5 | (2.8) | 0.6 | (-0.5 to 1.6) | 0.319 |

LDN, low dose naltrexone. DMARD, disease modifying antirheumatic drug. Three groups based on number of LDN dispenses: LDN ×1 ($N$ = 105) collected LDN once, LDN ×2–3 ($N$ = 75) two or three times and LDN ×4+ ($N$ = 180) four or more times. There were no users of anakinra, azathioprine, ciclosporin, mercaptopurine, rituximab, tacrolimus, and tocilizumab neither before nor after starting LDN.
